# Challenges faced by pediatric nursing workers in the face of the COVID-19 pandemic^*^

**DOI:** 10.1590/1518-8345.4550.3367

**Published:** 2020-09-07

**Authors:** Fernanda Garcia Bezerra Góes, Aline Cerqueira Santos Santana da Silva, Andressa Silva Torres dos Santos, Fernanda Maria Vieira Pereira-Ávila, Laura Johanson da Silva, Liliane Faria da Silva, Maithê de Carvalho e Lemos Goulart

**Affiliations:** 1 Universidade Federal Fluminense, Instituto de Humanidades e Saúde, Rio das Ostras, RJ, Brazil.; 2 Universidade Federal do Estado do Rio de Janeiro, Escola de Enfermagem Alfredo Pinto, UNIRIO, Rio de Janeiro, RJ, Brazil.; 3 Universidade Federal Fluminense, Escola de Enfermagem Aurora de Afonso Costa, Niterói, RJ, Brazil.

**Keywords:** Nursing, Pediatric Nursing, Nurse Practitioners, Coronavirus Infections, Coronavirus, Pandemics, Enfermería, Enfermería Pediátrica, Enfermeras Practicantes, Infecciones por Coronavirus, Coronavirus, Pandemias, Enfermagem, Enfermagem Pediátrica, Profissionais de Enfermagem, Infecções por Coronavírus, Coronavírus, Pandemias

## Abstract

**Objective:**

to identify the challenges pediatric nursing workers face as a result of the COVID-19 pandemic.

**Method:**

qualitative study, using a semi-structured electronic form applied to nursing workers from pediatric services in the state of Rio de Janeiro, Brazil. Data were submitted to lexicographic analysis using the *Interface de R pour Analyses Multidimensionnelles de Textes et de Questionnaires*, Word Cloud technique, and Similitude Analysis.

**Results:**

different challenges concerning the COVID-19 pandemic were reported, including the need to promote comprehensive and quality care while being concerned with protecting oneself and others, with an emphasis on fear. A lack of protective equipment, training, diagnostic tests, and knowledge/information concerning the disease was also reported, in addition to a reduced number of nursing workers and a lack of appreciation for the profession.

**Conclusion:**

managerial guidelines need to be adopted for properly allocating human and material resources in the health field, including the pediatric services, in addition to providing training on standard precautions. Actions to encourage, value, motivate, and support the nursing staff are needed during and after the pandemic to protect the physical and mental health of these professionals.

## Abstract (es)

**Objetivo:**

identificar los retos a los que se enfrentan los trabajadores de enfermería pediátrica como resultado de la pandemia de COVID-19.

**Método:**

estudio cualitativo, utilizando un formulario electrónico semiestructurado aplicado a trabajadores de enfermería de servicios pediátricos en el estado de Río de Janeiro, Brasil. Los datos fueron sometidos al análisis lexicográfico utilizando la *Interface de R pour Analyses Multidimensionnelles de Textes et de Questionnaire*, la técnica de *Word Cloud* y el Análisis de Similitud.

**Resultados:**

se informaron diferentes retos relacionados con la pandemia de COVID-19, incluida la necesidad de promover una atención integral y de calidad mientras preocupándose por protegerse a sí mismo y a los demás, con énfasis en el miedo. También se informó de la falta de equipo de protección, capacitación, pruebas diagnósticas e información sobre la enfermedad, además de un número reducido de trabajadores de enfermería y una falta de apreciación por la profesión.

**Conclusión:**

se deben adoptar directrices gerenciales para asignar adecuadamente los recursos humanos y materiales en el campo de la salud, incluidos los servicios pediátricos, además de proveer capacitación sobre las precauciones estándar. Se necesitan acciones para alentar, valorar, motivar y apoyar al personal de enfermería durante y después de la pandemia para proteger la salud física y mental de estos profesionales.

## Abstract (pt)

**Objetivo:**

identificar os desafios de profissionais de Enfermagem Pediátrica frente à pandemia da COVID-19.

**Método:**

pesquisa qualitativa, desenvolvida por meio de formulário eletrônico semiestruturado entre profissionais de Enfermagem que atuam em unidades pediátricas no Estado do Rio de Janeiro. Os dados foram submetidos à análise lexicográfica, com o auxílio do *software Interface de R pour Analyses Multidimensionnelles de Textes et de Questionnaires*, pelo método de Nuvem de Palavras e Análise de Similitude.

**Resultados:**

distintos desafios referentes à pandemia da COVID-19 foram relatados, dentre eles, a promoção de uma assistência integral e de qualidade frente à preocupação quanto à proteção de si e do outro, com destaque para o sentimento de medo. A falta de equipamentos de proteção individual, treinamentos, testes diagnósticos e conhecimentos/informações relacionados à doença, o número reduzido de profissionais de Enfermagem e a desvalorização da categoria também foram sinalizados.

**Conclusão:**

é primordial a adoção de diretrizes gerenciais para a adequada alocação de recursos humanos e materiais na área da saúde, inclusive, nos serviços pediátricos, incluindo treinamentos sobre as precauções-padrão. Além disso, são essenciais ações de incentivo, valorização, motivação e apoio à equipe de Enfermagem, durante e após a pandemia, para proteger a saúde física e mental desses profissionais.

## Introduction

The COVID-19 pandemic caused by the novel coronavirus (SARS-CoV-2) is an important public health crisis currently threatening humanity. The World Health Organization (WHO) reported more than 4,307,000 cases and 295,000 deaths worldwide up to mid-March 2020, and these numbers keep rising^(1)^. Up to the same period, Brazil recorded more than 202,000 cases with a lethality rate of 6.9%, ranking 6^th^ in the world in terms of the number of deaths^(2)^, higher than all developing nations.

Even though less vulnerable, children are not spared in this pandemic context. A systematic review reports that this group represents from 1% to 5% of the diagnostic cases and is more frequently associated with milder conditions when compared to adults, deaths being rare^(3)^. Children may be asymptomatic, though the elimination of the virus through respiratory secretion and feces seems to last longer than in adults, which may contribute to the spread of COVID-19^(4-5)^.

Children exhibit certain particularities and are unable to clearly describe their health conditions or report their history of contacts, which makes it a considerable challenge to protect, diagnose, treat and provide care to this population^(6)^. A review prior to the pandemic highlights that respiratory conditions such as pneumonia and asthma are important causes of hospitalization among Brazilian children^(7)^. The humoral and cellular immunity of children is not fully developed though, being unable to present an exacerbated inflammatory response. This may explain the peculiarity of children presenting relatively mild symptoms of COVID-19. It is also known that pediatric patients mainly belong to clustered cases, with one family contact confirmed with the disease, usually manifesting symptoms before the children^(8)^.

The most common symptoms among children include fever, dry cough, and fatigue in addition to nasal congestion, running nose, and sore throat. Severe pediatric cases present acute dyspnea and may rapidly progress to Acute Respiratory Distress Syndrome (ARDS), myocarditis, septic shock, refractory metabolic acidosis, coagulation dysfunction, and multiple organ failure^(4,8)^.

The largest pediatric series found was 2,143 children in China, with 34% of the cases confirmed through laboratory tests and 66% suspected cases. The median age was seven years old and 57% were boys. The proportion of asymptomatic, mild, moderate, and severe infections among the cases confirmed in the laboratory was 12.9%, 43.1%, 41%, 2.5%, and 0.4%, respectively^(9)^. Mild cases shall be monitored in primary health care centers and precaution measures implemented at home. Severe cases, however, shall be sent to a referral hospital for timely isolation and treatment^(10)^.

Children with a history of contacts with the severe form of COVID-19, with long-term use of immunosuppressant drugs, or younger than three months of age are the most vulnerable. Children with congenital malformations of heart, lungs, and airways, presenting chronic heart or kidney diseases, malnutrition, hereditary metabolic diseases, immunodeficiency, or cancer are likely to present the severe form of the disease and require hospitalization^(8)^. Hence, all hospitalized children with acute respiratory disease (fever, dry cough and/or shortness of breath), or asymptomatic children with direct contacts and those highly vulnerable with confirmed contact should be treated as suspected of being infected with COVID-19 in a hospital setting^(4)^.

Because the symptoms among children may not manifest as expressively as in adults, hospitalizations with respiratory symptoms may be confounded with other diseases and the measures necessary to prevent the spread of the virus may not be adopted. The consequences of a delayed verification of a patient with COVID-19 are considerable, especially for their contacts. Hence, health workers providing care to these children should be considered as highly vulnerable to exposure^(11)^. For this reason, pediatric facilities face unique challenges during this pandemic because, in addition to the fact that children with the infection present milder symptoms, they live with adults who may be infected, and usually are those who accompany or visit them during hospitalizations^(12)^.

The Brazilian Society of Pediatrics reinforces the need to implement measures to prevent the spread of COVID-19 among health workers in the services providing care to pediatric patients, which include: restricted visitation and/or accompaniment restricted to the primary caregiver, who should receive specific guidance on protective measures; all equipment necessary for protection has to be planned and provided, and a specific area needs to be designated for the provision of care to suspected or confirmed children^(13)^.

The nursing staff is in the front line dealing with this disease, working full time in the care provided to children and their families. Thus, even though these workers have invaluable information that can aid to understand the actual situation of pediatric health facilities in the face of the COVID-19 pandemic, studies addressing these workers were not found.

Concomitantly with the pandemic situation, the WHO designated 2020 as the “Year of the Nurse”, proposing a worldwide campaign called Nursing Now in partnership with the International Council of Nursing (ICN) and professional entities in various countries. This campaign is intended to highlight the role of nursing to achieve the health goals agreed by UN member countries, in addition to raising the status of nurses, considering their central role in the conception and implementations of health policies^(1)^. Hence, the value of the nursing work is doubly in evidence in times of pandemic.

Therefore, the nursing staff needs to have a voice so that public policies and institutional strategies are established to meet the real needs of workers, children, and families, envisioning the promotion of a safe and quality care delivery to all. Hence, this study’s objective was to identify the challenges faced by pediatric nursing workers in the face of the COVID-19 pandemic.

## Method

This descriptive and exploratory study with a qualitative approach was conducted using a semi-structured electronic form applied to nursing workers from pediatric units located in the state of Rio de Janeiro, Brazil. Inclusion criteria were: nursing workers (nurses and nursing technicians) who provided care in a hospital setting to individuals suspected of being infected or confirmed of having COVID-19.

Data were collected in the last half of April 2020 until theoretical saturation was achieved^(14)^, respecting the minimum necessary for analysis to be performed in the software, which recommends between 20 and 30 texts^(15)^. The workers were invited to participate in the study by two electronic media (e-mail or Whatsapp), and five days were established for the workers to return the completed instrument.

The snowball technique^(16)^ was used. It is a non-probabilistic, convenient sampling method, using reference chains to locate and recruit participants, and is mainly used for exploratory purposes and survey groups with difficult access given the pandemic, which restricts travels and contact among people. Hence, the first worker invited was nominated by the primary author and, later, the other co-authors, and the participants themselves provided the names of other potential participants at the end of the form.

A semi-structured form was developed and content validated by a panel of experts to collect data. The first part addressed information to characterize the participants including sex, age, profession, year of graduation, specialization, type of hospital, sector where the participant worked, and work shift. The second part included questions concerning their practice during the pandemic and the main challenges experienced in the period.

The completed forms were the primary source of data submitted to the lexicographic analysis using the *Interface de R pour Analyses Multidimensionnelles de Textes et de Questionnaires* (IRAMUTEQ)^(15)^, the Word Cloud technique and Similitude Analysis.

The National Commission on Research Ethics (CONEP) approved the study. All the participants were ensured that their identities and information provided would remain confidential, read an online free and informed consent form, and checked the option “I declare to have been informed and agree to participate as in this project a volunteer” to be included in the study. An alphanumerical code was used according to the order of participation.

## Results

Twenty-six (100%) nursing workers participated in the survey, most were women (96.1%), aged 33.1 years old on average, were nurses (84.6%), held a specialization degree (76.9%), in pediatric nursing (46.1%), and had graduated 12.3 years ago on average. Most participants worked in public hospitals (73.1%) located in the cities of Rio de Janeiro (65.3%), Niterói (19.2%), Duque de Caxias (7.7%), Rio das Ostras (3.8%), and São José do Vale do Rio Preto (3.8%) in the following wards: Pediatric Wards (57.7%), Pediatric Intensive Care Units (34.6%), Child Emergency (3.8%), and Pediatric Hematology (3.8%). Note that 38.8% of the workers did not report participation in COVID-19-related training programs though all participants reported having provided care to individuals suspected or confirmed being infected.

**Figure 1:**
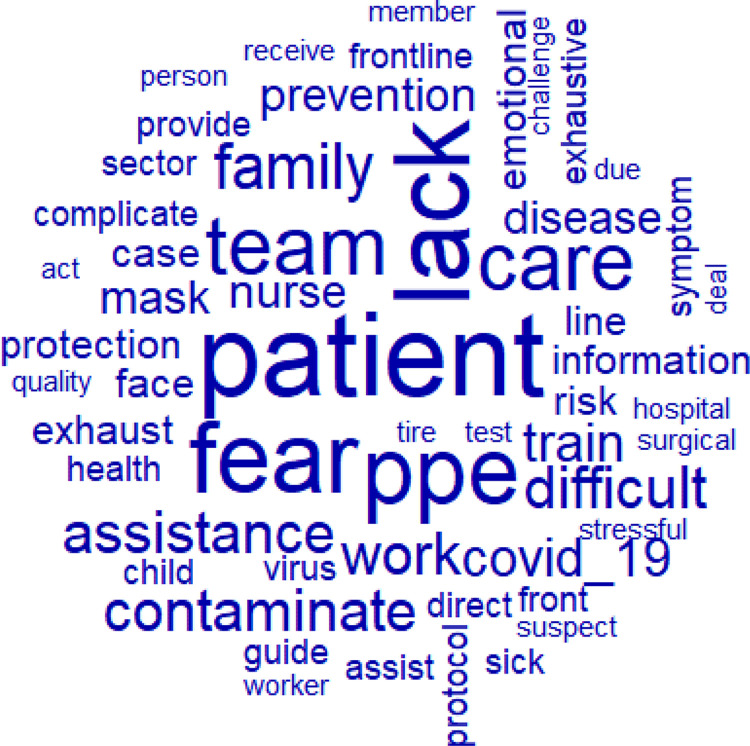
– Word cloud. Rio de Janeiro, RJ, Brazil, 2020

The *corpus* was composed of 26 texts, separated into 41 excerpts, with a total of 1,161 occurrences of words: 376 distinct words and 223 with a single occurrence (hapax). The Word Cloud analysis identified the words with the highest number of occurrences, and those with the highest frequency appear larger than the remaining words in Figure 1. The most recurrent active forms were: patient (18), lack (16), fear (15), PPE (14), team (12), and assistance (8).

The recurrence of these terms enabled identifying the main challenges faced by the pediatric nursing staff in the face of the novel coronavirus pandemic. The care provided to patients suspected or confirmed to be infected with COVID-19 is by itself considered a challenge. If, on the one hand, it requires the delivery of integral and quality care, on the other hand, it implies a great concern with one’s protection (health workers) and others’ protection (co-workers, the workers’ family members, children, companions, and other patients) to avoid the spread of the disease. Therefore, the terms “patient”, “assistance” and “contaminate” were frequently reported in the same excerpts.

*Providing integral assistance to patients, (...) in an attempt, preventing the contamination of others* (P8); *Promote quality assistance to patients and not compromise my health, or that of my co-workers, or that of my family. (...) Not contracting the virus by doing the best for patients and the team* (P10); *Not contaminating myself or my colleagues, providing the care necessary to heal patients* (P17); *Colleagues afraid of being contaminated but asymptomatic and transmit to other workers, non-COVID-19 patients, and companions, or our own families* (P18); *The work providing direct assistance to children in severe conditions, suspected or confirmed of having COVID-19. (...) There is little information at the time of a child’s hospitalization, whether she is suspected or not. For instance, a non-respiratory case that later worsens and tests positive. There are failures in the isolation and workers, children and families become exposed* (P16); *Not contaminating the team, patients or oneself* (P26); *monitor symptoms without compromising my individual protection, that of the health team and other patients* (P4); *Assist children and family members when they present symptoms of the disease. Guide families concerning prevention* (P23).

Another challenge nursing workers reported is related to the term “lack”, which also becomes evident in the word cloud, especially in terms of restricted personal protective equipment (“PPEs” was also a recurrent term and appear linked to the word “lack”) and other supplies necessary to work in the front line to combat this pandemic in pediatric units.

*Lack of proper PPEs. We work with N95 respirators that clearly are not sealed. There is a lack of surgical masks and aprons* (P14); *Fear of what lacks such as goggles, face shield* (P16); *I work in the nursing ward, the front line (...), lack of PPEs* (P1); *I’m in the front line providing direct care to patients with COVID-19 (...), lack of PPEs* (P12).

Concerning challenges, the word “lack” was also associated with lack of training, diagnostic tests, knowledge/information concerning COVID-19, as well as the decreased number of nursing workers and lack of appreciation of the profession.

*There is a lack of knowledge concerning the treatment. Lack of training* (P2); *Lack of training programs* (P14); *Very unprepared and lack of knowledge due to the rapid advancement of the disease* (P10); *Lack of workers and the profession is not appreciated* (P8); *Lack information concerning the disease. Lack of recognition and valorization* (P12); *The workers lack access to tests* (P18).

Note that despite individuals’ concern regarding protection against the disease, the workers, especially the nurses, highlighted challenges concerning team management, whether reporting an insufficient number of workers or the need to provide COVID-19-related guidance and training to workers.

*Teams working with a minimum number of workers* (P12); *Taking continuous education programs regarding COVID-19 with my teammates* (P14); *Protective clothing makes the team feel insecure (...). Guidance and training to the team* (P18); *Develop flowcharts, provide direct care to patients, train the team* (P22); *Guide the other nursing team members regarding prevention and protection, while there are cases in the unit* (P23).

Amidst these challenges, “fear” was the emotion most evident in the participants’ responses, a constant feeling among pediatric nursing workers, as they reported being afraid of contaminating themselves and their families.

*We are afraid for ourselves and our families* (P4); *Especially, fear of being infected by the coronavirus* (P7); *Fear of contaminating you and your family* (P8); *there is a constant fear of taking it home and contaminating my family* (P11); *Everyone is afraid of contracting the disease* (P12); *Fear of getting contaminated and contaminate your loved ones (...) fear of becoming sick* (P23).

Note that the words “difficult” (6), “exhausting” (5), “tense” (4), “weary” (4) and “stressful” (3) were also present in the workers’ answers when they reported the main challenges nursing workers experienced at this time.

**Figure 2:**
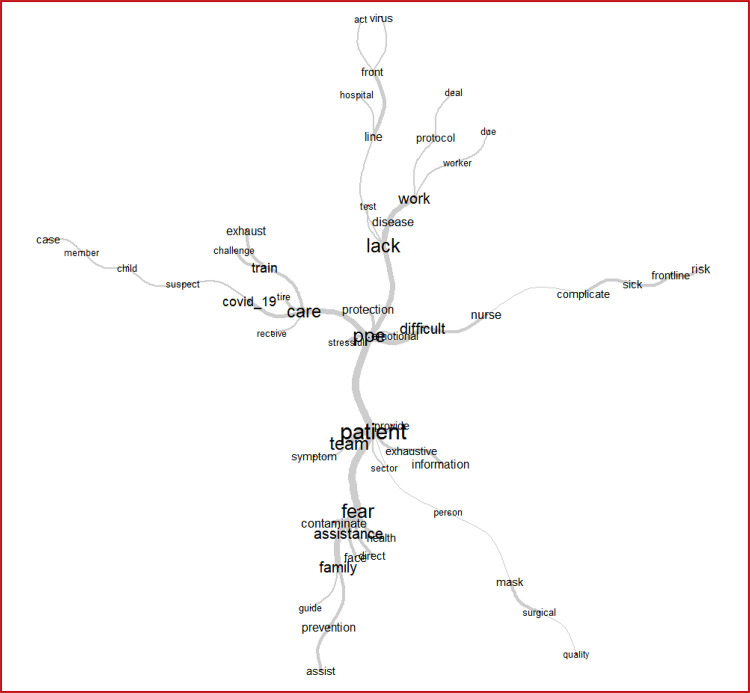
– Similitude analysis. Rio de Janeiro, RJ, Brazil, 2020

The synthesis of these challenges reported by the pediatric nursing workers may be better understood through the graphic analysis of similitude (Figure 2), which shows the connectivity between words with the most recurrent ones. The most frequent ones appear larger to aid the identification of the structure of the *corpus* content. For instance, the word “fear” and its proximity with “assistance” is highlighted, as well as the connection between the terms “lack” and “PPEs”, reinforcing the findings presented thus far.

## Discussion

The study’s findings show different challenges concerning the COVID-19 pandemic from the perspective of pediatric nursing workers, with an emphasis on the promotion of integral and quality care while at the same time being concerned with protecting oneself and others, with an emphasis on fear.

The health sector is facing a pandemic of a highly transmissible and mortal disease in real time and, at the same time, has to deal with the fragility of the health system in the provision of sufficient equipment and basic protection supplies. Thus, the concern the participants report is legit as workers are very likely to develop the disease in addition to facing other problems of a psychological nature that accrue from facing challenges imposed by the COVID-19 pandemic^(17)^.

A survey conducted in China confirmed that hospitals are the main sources of secondary transmissions, reporting 1,716 cases, and five deaths among health workers up to February 2020^(18)^. In Brazil, according to a balance reported by the Federal Council of Nursing (COFEN), at least 108 deaths of nursing workers were caused by COVID-19 up to mid-May 2020, in addition to more than 10,000 workers on sick leave, either suspected or confirmed of having the disease. Additionally, there were more than 5,000 complaints concerning a lack of or a restricted number of PPEs^(19)^. Hence, it is imperative to protect oneself and others to contain the spread of the disease within hospital settings, which is in line with current findings.

It is important to reinforce that, even though children seem to present milder forms of COVID-19, they are not immune to contamination^(18)^. Note that approximately 20% of the cases among children are asymptomatic, which brings up the potential transmissibility of the novel coronavirus, considering the close contact between children with the virus and health workers and their families^(20)^.

Being in a constant state of alertness, fear, and tension, and having the responsibility to provide harm-free and quality care despite limited resources, in a context of continuous exposure and uncertainty of whether one has been contaminated or not, configure elements that contribute to both physical and mental burnout^(21)^. In this sense, we reinforce the need for managers to be proactive in terms of promoting the wellbeing of workers^(17)^.

Another study highlights that the workers’ fear of becoming a carrier of the virus and transmit it to co-workers and their own families has led to high levels of psychological stress^(22)^. Such a fear is justified due to cases of asymptomatic transmission of COVID-19^(11)^, as it happens among children.

The scientific literature reinforces that experiences of pain, suffering, and death, associated with intense work rhythms, long working hours without rest, low salaries, complex human relationships, and a lack of supplies and human resources, are stressful factors that may lead not only to illness but mainly to fear^(23)^, which is in line with the findings of the lexicographic analysis presented here.

Therefore, the workers’ adaptation in the face of COVID-19 has led to drastic changes in the workplace and can be seen as one of the main challenges faced in this pandemic^(24)^. As reported by the pediatric nursing workers, this adaptation implies experiencing fear, exhaustion, tension, weariness, and stress in the face of the possibility of contagion/infection and death caused by the novel coronavirus, which corroborates with the literature^(25)^.

For these professionals to work while dealing with fear due to the high likelihood of being contaminated, continuous training addressing standard precautions is essential as well as to provide supplies, so that workers recognize the dangers and adopt a safe behavior. Additionally, the inclusion of topics such as the planning of protective measures at home and with family members, such as taking the shoes off, removing and washing clothes, and taking a shower immediately after arriving home, among other measures, can alleviate stress and anxiety^(11)^.

Emotional issues are enhanced by difficulties in managing the crisis which the pandemic imposes on governments, public policymakers, local managers, and teams in health units. The health needs this pandemic creates surpass the capacity of hospitals and health systems, leading to a stressful scenario for health workers who are in front line services. The reason is that care involves the emergency management of processes as well as material and human resources, which, as reported by the literature, has been an important challenge faced worldwide^(26)^.

In this sense, the work context of these professionals is marked by a scarcity of PPEs, lack of training, and reduced human resources, which increases the fear of becoming contaminated in a pediatric unit when in contact with team members, children and families, and of becoming vectors, contaminating their families at home. It is known that labor conditions involving aspects such as safety, influence one’s emotional response and communication ability in the patient-nurse relationship^(27)^.

Concerning material resources, the participants’ reports show a crisis related to the provision of quality of the material that is essential to prevent and combat the disease such as diagnostic tests and PPE, directly impacting patient safety and health practice. This is a crisis various countries face and which demands leadership at the various managerial levels, as well as a just and ethical allocation of health resources in the face of imbalances between supply and demand during the COVID-19 pandemic. Thus, priorities need to be established in the allocation of resources so that health workers are not required to make isolated decisions that may be traumatic emotional terms^(28)^.

This study’s participants reported challenges closely related to the management of material resources, that is, related to human resources, especially in terms of a decreased number of nursing workers. This situation may be related to an insufficient number of workers even before the pandemic and that was aggravated by the recent leave of workers.

The need to immediately remove asymptomatic health workers who have contact at home with individuals suspected or confirmed of being infected and workers suspected of having the flu syndrome (fever accompanied of cough or sore throat or difficulty breathing), coupled with the leave of more vulnerable workers, considerably decrease the number of teams and overload those who remain working^(29)^. Hence, it is a challenge to balance the equation between seven to 14-day sick leaves with the increased demand in health services.

Other challenging situations in human resources that were highlighted in this study concerned a lack of knowledge/information regarding the disease, the urgent need for specific guidance and training, and a perception of a lack of appreciation of the profession; the latter is a recurrent complaint of those who constitute the largest profession in the health field^(30)^.

Similar challenges faced in a tertiary hospital in Wuhan, China triggered emergency responses, especially related to the work of nurses. Thus, strategies were implemented to manage the nursing workforce, involving significant hospital mobilization promoted by the leadership to provide training, supervision, and adjust priorities in the local context. Interesting measures were adopted to encourage, value, and motivate workers and support those in the front line, including messages sent through mobile phones and the dissemination of achievements on an official website^(31)^.

Thus, the COVID-19 pandemic presents a great opportunity to assess the health systems. From this perspective, considering that 2020 is the “Year of the Nurse”, being amidst this pandemic does not exclude the importance of establishing a political discussion of nursing. On the contrary, this is a time to show the capacity of work and the real needs of these workers to outline appropriate strategies to face this and other pandemics in the future^(32)^.

This study’s limitations are related to the need to offer the participants an electronic form, resulting in the participants providing more objective answers when compared to a face-to-face interview, which was unfeasible during the period of data collection due to social isolation measures.

Despite these limitations, this study presents important contributions to the advancement of scientific knowledge related to the combat of the pandemic by pediatric nursing workers in the Brazilian context, as it shows important objective issues related to difficulties concerning the training and management of human and material resources, as well as to these workers’ emotional subjectivity, revealing important emotional load among those working in the front line.

## Conclusion

Given the COVID-19 pandemic, the pediatric nursing workers reported different challenges, with an emphasis on the promotion of integral and quality care while being concerned with protecting oneself and others. Being afraid of becoming infected and infecting family members was the feeling most frequently reported.

The challenging context reported in this study encourages reflections on the potential impact of emotional burnout, apparent in the stress and fear reported by the nursing workers in the front lines to combat this pandemic. It is believed that this heightened perception of insecurity may influence the discontinuity of humanized practices in the care provided in the context of pediatric hospitals, which in turn deserves investment in future research.

Additionally, the participants reported a lack of PPEs, training, diagnostic tests as well as of knowledge/information concerning the disease, in addition to a reduced number of nursing workers and non-appreciation of the profession, showing the urgent need to provide guidance and specific training.

The scarcity of resources necessary to provide proper care requires reflections on the imperative need for management guidelines to determine the allocation of resources, considering the context and realities of the different care settings and the pace at which the pandemic develops. Nursing leaders need to devise strategies, also for pediatric services.

Finally, the management of continuous education addressing COVID-19 among nursing workers, implementing institutional protocols, should involve training on standard precautions, aiming to provide safe care; and should address behavioral elements to strengthen teamwork and interaction to provide education to family members accompanying their children during hospitalization. It is also crucial that managers adopt measures to encourage, value, motivate, and support the nursing staff during and after the pandemic to protect these workers’ physical and mental health.
